# The Influence of Life History on the Response to Parasitism: Differential Response to Non-Lethal Sea Lamprey Parasitism by Two Lake Charr Ecomorphs

**DOI:** 10.1093/icb/icac001

**Published:** 2022-01-13

**Authors:** Tyler J Firkus, Frederick W Goetz, Gregory Fischer, Cheryl A Murphy

**Affiliations:** Department of Fisheries and Wildlife, Michigan State University, East Lansing, MI 48824, USA; Great Lakes WATER Institute, University of Wisconsin-Milwaukee, Milwaukee, WI 53204, USA; Northern Aquaculture Demonstration Facility, University of Wisconsin-Stevens Point, Bayfield, WI 54814, USA; Department of Fisheries and Wildlife, Michigan State University, East Lansing, MI 48824, USA

## Abstract

The energetic demands of stressors like parasitism require hosts to reallocate energy away from normal physiological processes to survive. Life history theory provides predictions about how hosts will reallocate energy following parasitism, but few studies provide empirical evidence to test these predictions. We examined the sub-lethal effects of sea lamprey parasitism on lean and siscowet lake charr, two ecomorphs with different life history strategies. Leans are shorter lived, faster growing, and reach reproductive maturity earlier than siscowets. Following a parasitism event of 4 days, we assessed changes to energy allocation by monitoring endpoints related to reproduction, energy storage, and growth. Results indicate that lean and siscowet lake charr differ considerably in their response to parasitism. Severely parasitized leans slightly increased their reproductive effort and maintained growth and energy storage, consistent with expectations based on life history that leans are less likely to survive parasitism and have shorter lifespans than siscowets making investing in immediate reproduction more adaptive. Siscowets nearly ceased reproduction following severe parasitism and showed evidence of altered energy storage, consistent with a strategy that favors maximizing long-term reproductive success. These findings suggest that life history can be used to generalize stressor response between populations and can aid management efforts.

## Introduction

Parasitism is an energetically costly stressor for hosts. Coping with the energetic demands of parasitism necessitates diverting energy away from other physiological processes such as growth and reproduction, and results in alterations to host physiology and behavior (Barber et al. 2000; Barber 2007; Iwanowicz 2011; Allan et al. 2020). In many studies involving fish under the stress of parasitism, energy is redirected from reproduction and invested in processes that mitigate negative influences on survival such as growth or immunity (Lemly and Esch 1984; Adlerstein and Dorn 1998; Hecker and Karbe 2005). In other cases, stressor-induced energy limitation can result in the maintenance of reproduction at the expense of survival (Agnew et al. 2000; Wingfield and Sapolsky 2003). The most advantageous response in the face of parasitism-driven energy limitation depends on the life history of the host and the specific stress mechanism of the parasite (Forbes 1993; Agnew et al. 2000; Alvestad 2017). Hosts that are longer lived and have many opportunities to reproduce during their lifetimes, are likely to maximize fitness by diverting resources away from reproduction in the short-term, if by doing so it allows the host to have more opportunities to reproduce in the future. This strategy is only adaptive if the likelihood of surviving parasitism is high, and parasitism does not lead to a future reduction in the ability to reproduce (Forbes 1993). Conversely, if a host has a relatively short lifespan and fewer opportunities to reproduce during its lifetime, diverting resources away from reproduction may not increase fitness as there are fewer opportunities to later compensate for the loss of reproductive effort. These adaptive responses to parasitism are well-grounded in life history theory (Forbes 1993; Agnew et al. 2000), but robust empirical evidence addressing these responses is lacking for most animals (Valenzuela‐Sánchez et al. 2021).

A particularly good case for examining life history trade-offs experienced in the face of parasitism is the interaction between lake charr (*Salvelinus namaycush*) and the invasive sea lamprey (*Petromyzon marinus*) in the Laurentian Great Lakes. Lake charr in the Laurentian Great Lakes display considerable variability influenced by environmental adaptation, with four currently recognized lake charr ecomorphs differing in appearance, habitat preference, and life history characteristics (Moore and Bronte 2001; Muir et al. 2014; Hansen et al. 2016; Sitar et al. 2020). Lake charr are a preferred host species for sea lamprey in the Laurentian Great Lakes (Harvey et al. 2008; Johnson et al. 2021). Although lake charr mortality following sea lamprey parasitism occurs frequently, an estimated 45–75% of lake charr survive sea lamprey parasitism events (Swink 2003; Madenjian et al. 2008). Lake charr that survive sea lamprey parasitism often make up a large proportion of the total population; evidenced by high rates of lake charr with sea lamprey wounds (Sitar et al. 1997; Rogers et al. 2019). However, little is known about lake charr that survive sea lamprey parasitism. In the short-term, sea lamprey parasitism alters host lake charr plasma sex steroid concentrations (Smith et al. 2016), blood chemistry (Edsall and Swink 2001), plasma protein expression (Bullingham et al. 2021), and transcriptional regulation of genes involved in inflammation, cellular damage, and energy utilization (Goetz et al. 2016). In the longer term, parasitism influences expression of proteins related to immune response, lipid transport, and blood coagulation (Bullingham et al. 2021). It is possible that these health repercussions could lead to a diversion of energy from normal physiological processes such as growth, immune function, and reproduction, and have long-term implications. Furthermore, the response to parasitism may vary depending on the life history characteristics of the lake charr ecomorph that has been parasitized (Smith et al. 2016).

Siscowets and leans are two lake charr ecomorphs found in Lake Superior, that have considerable life history and morphological differences that could influence their response to parasitism. Leans prefer shallower depths and warmer water temperatures, have relatively low muscle lipid content, and are faster growing than their siscowet counterparts (Moore and Bronte 2001; Sitar et al. 2020; Chavarie et al. 2021). Siscowets generally live deeper in the water column and experience cooler water temperatures (Moore and Bronte 2001; Chavarie et al. 2021; Jasonowicz et al. submitted for publication). As a result, siscowets are slower growing and reach reproductive maturity much later than leans (Sitar et al. 2014). A high proportion of siscowets also do not put forth a reproductive effort each year (“skipped spawning”). Although leans also display skipped spawning, they do so less frequently (∼12% for leans and ∼58% for siscowets; Sitar et al. 2014). Siscowets tend to have a higher rate of observed sea lamprey parasitism in the Laurentian Great Lakes than leans, suggesting a higher % survive sea lamprey attacks (Bence et al. 2003; Horns et al. 2003; Sitar et al. 2008; Moody et al. 2011). Survival may be higher for siscowet lake charr because of the cooler water temperatures they inhabit, which would reduce sea lamprey metabolism resulting in less severe attacks (Bence et al. 2003; Sitar et al. 2008), though identifying the mechanism driving survival differences among ecomorphs requires further exploration. Additionally, siscowets appear to cope better with parasitism than leans as they do not show altered growth trajectories following parasitism (Smith et al. 2016). The two ecomorphs also show molecular and physiological differences following parasitism that suggest siscowets buffer critical physiological processes important for survival (Sitar et al. 2008; Goetz et al. 2016; Smith et al. 2016).

The objective of this study was to evaluate the long-term effects of sea lamprey parasitism on reproduction, growth, and energy storage in two lake trout ecomorphs with differing life histories. The short-term impacts of sea lamprey parasitism have been studied, but it is uncertain if those identified impacts have long-term consequences. We approached this by assessing the sublethal effects of sea lamprey parasitism on growth, energy storage, and the reproductive physiology of siscowet and lean lake charr and comparing the findings with expectations from our conceptual model. We experimentally allowed sea lamprey to parasitize siscowet and lean lake charr that were raised in a common environment and monitored long-term effects on reproductive endpoints. If life history plays an important role in dictating how parasitism stress is addressed, we expected these two ecomorphs to display key differences in their parasitism response that are consistent with expected optimal strategies (Table 1). Briefly, because siscowets are longer lived and less likely to die following parasitism, we expect them to respond to sea lamprey parasitism by diverting energy away from reproduction in the short-term to increase survival, maintain critical lipid reserves, and maximize future reproductive success. Because leans are shorter-lived and less likely to survive parasitism, we expected to observe comparatively less diversion of energy away from reproduction following sea lamprey parasitism as there is less benefit to increasing future reproductive success at the expense of current reproduction. Leans may instead compensate by allocating energy away from growth and storage, and toward surviving the parasitism event and maintaining reproduction. A better understanding of the parasite–host relationship and how it differs between life history strategies, could assist in generalizing responses to stressors and to assist management efforts.

**Table 1:** Conceptual model of expected relative energy allocation in siscowet and lean lake charr with and without sea lamprey parasitism

Energetic category	Morphotype	Energy allocation	Change in energy allocation under parasitism
**Growth**	Lean	High	Decrease
	Siscowet	Low	Decrease
**Storage**	Lean	Low	Decrease
	Siscowet	High	Maintain
**Reproduction**	Lean	High	Maintain
	Siscowet	Low	Decrease

## Material and methods

### Study organisms

We used 11–12 year-old siscowet (*n* = 82, 1.93–4.78 kg) and lean (*n* = 89, 1.81–5.25 kg) lake charr ecomorphs raised from eggs collected from wild adult lake charr in Lake Superior in the autumn of 2006. The lake charr were raised in identical laboratory conditions (1.5 × 21 m raceways, 6.8–8.3°C, natural photoperiod), and maintained the morphometric, physiological, and life history differences expected of wild siscowet and lean lake charr (Goetz et al. 2010, 2014). Lake charr were fed a maintenance diet (0.5%) of Rangen 8.0 mm EXTR 450 Trout Feed (Buhl, ID), and excess uneaten food was observed at each feeding. No feed was provided during parasitism trials. Each lake charr had an implanted pit tag allowing for individuals to be tracked.

Actively parasitic sea lamprey used in this study were collected from wild lake charr hosts by commercial fishing operations in summer and early-autumn of 2016 and 2017 from Lake Superior and Lake Huron. Sea lamprey (*n* = 44, 44–241 g) were screened for disease prior to transfer into the lab and were kept in flow-through tanks isolated from the lake charr when not in-use for parasitism trials.

### Parasitism trials

Parasitism trials were conducted during November through December of 2016 and 2017 following Michigan State University Institutional Animal Care and Use Committee approved protocols. Briefly, individual lake charr were placed in separate 1000 L circular tanks, containing one sea lamprey. Tanks were checked three times daily, and the time of sea lamprey attachment was recorded. Sea lamprey were removed after 4 days of feeding to prevent lethal parasitism (Smith et al. 2016). An additional group of lake charr were individually placed in the 1000 L tanks without sea lamprey for a similar duration as the parasitism trials to serve as controls.

**Fig. 1:**
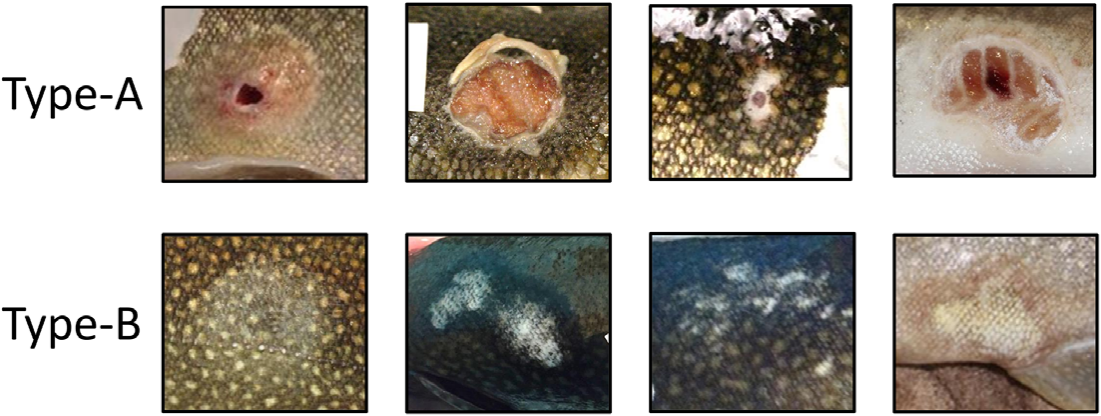
Examples of difference in parasitism severity of type-A and type-B wounds.

Immediately following each parasitism trial, length, weight, and fat content of the lake charr was measured, and the resulting wound was classified using current sea lamprey wound classification guidelines (Ebener et al. 2006). Because the severity of sea lamprey parasitism varies considerably, natural resources agencies in the Great Lakes routinely survey lake charr hosts and score observed sea lamprey wounds using a binary classification system that associates wound characteristics with the severity of the parasitism event. A severe wound is classified as type-A and is recorded when the skin at the wound site is broken and the underlying musculature exposed. Type-B wounds are less severe and are recorded when the wound site was abraded (scales removed), but the skin was not broken (Fig. 1). These wound types were considered separately because the severity of the parasitism event is likely to influence the magnitude of the response to parasitism. After wound assessment, lake charr were transferred back to their raceways and allowed to heal.

### Endpoints of interest

To assess changes into growth, length, and weight, lake charr were measured in October prior to parasitism trials, and in October the year following parasitism trials. Each lake charr was removed from the raceways and anesthetized with tricaine methane sulfonate (MS-222). Excess water was blotted from each lake charr with paper towels before total length (to nearest cm) and weight (to nearest 10 g) were recorded. The difference in length and weight between these time points was used to represent alterations to growth.

Alterations to energy storage were measured by assessing the change in muscle lipid concentrations and the hepatosomatic index (HSI) in August following parasitism trials. For the change in muscle lipid concentration, each lake charr was measured with a handheld microwave fatmeter (Distell Inc., Model FFM-692, West Lothian, Scotland) immediately after length and weight measurements. Lipid concentrations were again measured in October prior to parasitism trials, and in October following parasitism trials, and the difference between these time points was used. To obtain HSI, an indicator of energy storage (Skjæraasen et al. 2012; Goetz et al. 2014; Sitar et al. 2014), a subset of lake charr were lethally sampled in August following parasitism trials. We sampled 31 siscowets—17 females (five type-A wounded, five type-B wounded, and seven control) and 14 males (three type-A wounded, four type-B wounded, and seven control)—and 33 leans—16 females (three type-A wounded, five type-B wounded, and eight control) and 17 males (five type-A, seven type-B, and five control). These lake charr were not used for reproduction or growth analysis. Lake charr were euthanized with an overdose of MS-222, and livers were surgically extracted and weighed. HSI was calculated as $\frac{{liver\ weight}}{{body\ weight}}x\ 100$.

Reproduction was measured by obtaining the normalized egg mass, skipped spawning (females), and milt sperm cell concentration (males). Both ecomorphs generally spawn in early October, so from mid-September through early-November, we regularly monitored each lake charr for ovulation and spermiation. Sample sizes for reproductive measurements were: 51 siscowets—29 females (nine type-A wounded, 10 type-B wounded, and 10 control) and 22 males (seven type-A wounded, seven type-B wounded, and eight control)—and 57 leans—31 females (10 type-A wounded, 10 type-B wounded, and 11 control) and 26 males (seven type-A wounded, nine type-B wounded, and 10 control). Any lake charr ready to spawn were stripped of eggs or milt. The total volume of eggs stripped and egg mass was collected from each female. To account for size-related difference in egg production, egg mass was standardized by dividing by the wet weight of each individual female. Females that did not produce any eggs were deemed to have skipped spawning (Sitar et al. 2014).

A total of four 15 mL samples of eggs from each spawning female were kept for fertilization trials to assess embryo survival. A total of two samples were fertilized with milt from an individual parasitized male and two samples were fertilized with milt from an individual control male. Embryos were monitored weekly, and the proportion surviving to swim-up were recorded.

### Additional co-variates

Because we were interested in other factors that may contribute to growth, energy storage, and reproduction in addition to sea lamprey parasitism, we collected additional information from lake charr at various time points. At the beginning of each month from July through October following parasitism, we took additional sub-lethal samples measuring length, weight, condition factor, muscle lipid concentration, plasma estradiol and testosterone concentrations, and hematocrit. A 0.5 mL blood sample was taken *via* heparinized syringe from the caudal vein and centrifuged to separate plasma and packed red blood cells. Each fraction was stored separately at −80°C before plasma sex steroid concentrations were assessed using radioimmunoassays (raw steroid profiles are included in Supplementary Figs. S2 and S3). Additional blood was collected in a hematocrit tube, centrifuged, and blood hematocrit (ratio of packed red blood cell volume to total volume) was recorded. Following sampling, each fish recovered in a MS-222 free holding tank and then returned to the raceways. These additional data were considered as potential co-variates when estimating the influence of parasitism on our endpoints of interest. Mean values of endpoints of interest and selected co-variates separated by ecomorph and parasitism status are included in Table 2.

**Table 2:** Mean values (and standard deviations) of endpoints of interest and selected co-variates separated by ecomorph and parasitism status (type-A, type-B, and control).

			Means (SD)		
Measurement	Month measured	Ecomorph	Type-A	Type-B	Control
Sample size	August post-parasitism	Lean	8	12	13
		Siscowet	8	9	14
	October post-parasitism	Lean	17	19	21
		Siscowet	16	17	18
Change in length (cm)	October post-parasitism	Lean	1.9(6.3)	4.1(6.1)	3.9(4.5)
		Siscowet	1.81(3.12)	−1.94(18.7)	0.8(6.7)
Change in weight (kg)	October post-parasitism	Lean	−0.04(0.2)	−0.05(0.3)	−0.01(0.2)
		Siscowet	−0.11(0.19)	−0.35(0.42)	−0.15(0.26)
Change in muscle lipid (%)	October post-parasitism	Lean	2.27(7.45)	1.17(6.53)	2.8(8.0)
		Siscowet	0.08(7.55)	−0.68(4.73)	1.67(5.22)
HSI	August post-parasitism	Lean	1.06(0.31)	1.08(0.47)	1.18(0.36)
		Siscowet	1.21(0.49)	1.14(0.32)	1.00(0.28)
Standardized egg weight	October post-parasitism	Lean	0.11(0.02)	0.11(0.02)	0.10(0.02)
		Siscowet	0.02(0.04)	0.04(0.04)	0.04(0.04)
Skipped spawning (female only; %)	October post-parasitism	Lean	0	0	0
		Siscowet	78	30	50
Initial muscle lipid (%)	October pre-parasitism	Lean	21.58(9.86)	20.10(4.63)	21.27(9.64)
		Siscowet	51.61(7.05)	50.83(4.28)	51.74(6.12)
Initial weight (kg)	October pre-parasitism	Lean	3.15(0.87)	3.16(0.65)	3.21(0.87)
		Siscowet	3.41(0.66)	3.59(0.58)	3.31(0.79)
Initial length (cm)	October pre-parasitism	Lean	690.8(45.7)	702.1(48.4)	705.9(53.5)
		Siscowet	710.4(43.3)	718.4(41.3)	694.1(48.3)
Estradiol concentration (female only; ng/mL)	September post-parasitism	Lean	12.3(3.06)	7.63(3.28)	8.71(4.35)
		Siscowet	8.70(5.26)	9.82(7.33)	8.35(4.85)
Testosterone concentration (female only; ng/mL)	September post-parasitism	Lean	23.7(10.8)	27.17(13.49)	28.81(15.54)
		Siscowet	23.04(15.90)	18.23(9.15)	26.34(11.06)
Condition factor	October pre-parasitism	Lean	0.98(0.13)	0.94(0.11)	0.90(0.08)
		Siscowet	0.97(0.06)	1.00(0.10)	1.00(0.09)

### Analysis

We were interested in the influence sea lamprey parasitism had on growth, storage, and reproductive outcomes for siscowet and lean lake charr. To assess growth, changes in length and weight were used as endpoints of interest. HSI and muscle lipid concentration were used to assess effects on storage. To assess impacts on reproduction, egg production, milt production, and skipped spawning were used as endpoints. Because there may be several factors that contribute to growth, storage, and reproductive success besides parasitism (additional co-variates), we used Bayesian multiple linear regression to evaluate competing models for each endpoint of interest. This approach is critical as it allows us to assess the influence of sea lamprey parasitism while accounting for other important processes that may also play a role. For each endpoint of interest, we developed a list of plausible *a priori* candidate models that could best explain what drives any observed changes. Models were run separately for each ecomorph. Models were fit using JAGS (Plummer 2016) using the jagsUI package (Kellner 2019) with R version 3.6.1 (R Core Team 2019). Each model was fitted with diffuse flat priors on the model parameters, a choice made to be weakly informative, and using three Markov chains, 100,000 iterations, a burn-in of 20,000, and a thinning rate of 2. Posterior distributions were assessed for convergence with the Brooks–Gelman–Rubin statistic ($\hat{R}$ values < 1.1 indicated convergence) (Brooks and Gelman 1998) and with visual assessment of posterior distributions. The resulting chains all had effective sample sizes > 10,000 for all variables, indicating they provided a good characterization of the posterior distribution. The *a priori* candidate models for each endpoint of interest were ranked using the deviance information criterion (DIC), with the lowest DIC indicating the most parsimonious model. We assumed that models with ΔDIC < 2 were plausible. Credible intervals were obtained from posterior estimates of parameter values (all three chains combined), and only model parameters with 90% credible intervals (90%CrI) that did not overlap zero were considered (Spiegelhalter et al. 2002).

## Results

### Growth

Parasitism did not influence growth as measured by change in length for lean or siscowet lake charr. None of the best performing models for change in length (Supplementary Table S1) had parameters with 90% credible intervals that did not contain zero. This ultimately indicates that there were no differences in growth that could be explained by our co-variates. Similarly, parasitism did not influence the change in weight following parasitism for lean or siscowet lake charr. The best performing models for change in weight all had parameters with 90% credible intervals containing zero.

### Energy storage

**Fig. 2:**
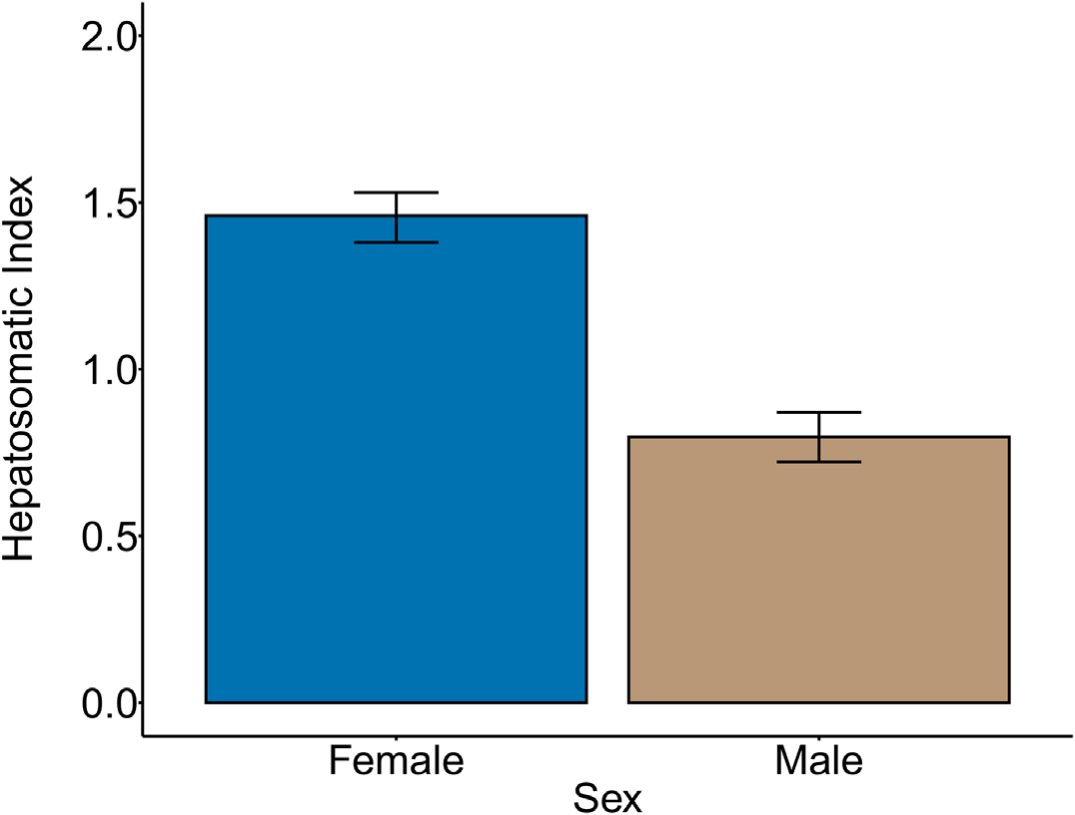
Marginal effect of sex in the most parsimonious model estimating HSI for lean lake charr. The height of each bar indicates the posterior mean, and error bars represent 90% credible intervals.

**Fig. 3:**
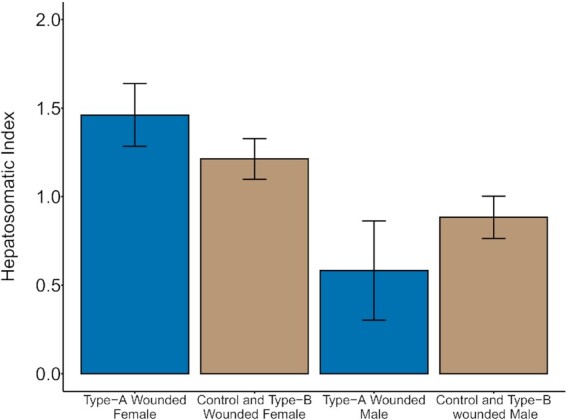
HSI estimates from the most parsimonious model for siscowet lake charr. The height of each bar indicates the posterior mean, and error bars represent 90% credible intervals.

Severe parasitism affected the HSI of female and male siscowet lake charr, but not lean lake charr of either sex. For lean lake charr, HSI was only influenced by sex. Female leans had HSI values 0.66 (90%CrI = 0.54–0.78) higher than male leans (Fig. 2). Models including parasitism status did not perform within 2 DIC of the sex only model (Supplementary Table S1). For siscowet lake charr, the best model included presence of a type-A wound, sex, and an interaction between the two. Female siscowets had HSI values 0.33 (90%CrI = 0.14–0.51) higher than siscowet males. The effect of a type-A wound depended on sex. For females with type-A wounds, HSI values were 0.55 (90%CrI = 0.14–0.96) higher than for control and type-B wounded females. For males with type-A wounds, HSI values were 0.30 (90%CrI = −0.64–0.04) lower than control and type-B wounded males, but the 90% credible interval contained zero (Fig. 3). A total of two additional models had DIC values within 2 of the best performing model (Supplementary Table S1). One had the addition of the presence of a type-B wound, but the 90% credible interval contained zero. The other model included only sex as a predictor, similar to leans. In this model, female HSI values were 0.45 higher for than for males (90%CrI = 0.28–0.62).

Changes in muscle lipid concentration following parasitism was not influenced by parasitism for lean lake charr. None of the models estimating change in muscle lipid in the year following parasitism for leans had parameter estimates with 90% credible intervals not containing zero. For siscowet lake charr, muscle lipid was influenced by sex, but not parasitism. Female siscowets gained 5.16% less muscle lipid (90%CrI = −7.70 to −0.63) than male siscowets. No other models had DIC values within 2 of the sex-only model (Supplementary Table S1).

### Egg production

For lean lake charr, both severe parasitism and the change in length in the year following parasitism influenced egg production (Supplementary Table S1). Leans with a type-A wound were associated with a 0.019 g (90%CrI = 0.005–0.032) increase in egg weight per g of body weight relative to control and type-B wounded leans. A 1 mm increase in length in the year following parasitism was associated with a 0.002 g (95%CrI = 0.001–0.003) increase in egg weight per g of body weight for leans. For the average weight lean lake charr in our sample (3.05 kg), a type-A wound would be associated with a 58 g increase in egg mass (17% of the average egg mass for lean females), and a 2.7 cm increase in length (average for lean females) is associated with a 16.4 g increase in egg mass (5% of the average egg mass for lean females).

For siscowet lake charr, parasitism did not influence egg production as measured by egg weight standardized to body weight. However, the muscle lipid concentration prior to parasitism did affect egg production. The best performing model included the presence of a type-A wound and the % muscle lipid content prior to parasitism, but only the % muscle lipid content had a 90% credible interval that did not contain zero (Supplementary Table S1). Siscowets with type-A wounds were associated with a 0.02 g decrease in egg weight per g of body weight relative to control and type-B wounded siscowets, however the 90% credible interval contained zero (90%CrI = −0.043–0.002). A 1% increase in initial muscle lipid content was associated with a 0.003 g (95%CrI = 0.001–0.005) increase in egg weight per g of body weight. There were four additional models that had DIC values within 2 of the best performing model (Supplementary Table S1), and these models contained a combination of initial muscle lipid, presence of a type-A wound, September E2 concentration, and presence of a type-B wound.

**Fig. 4:**
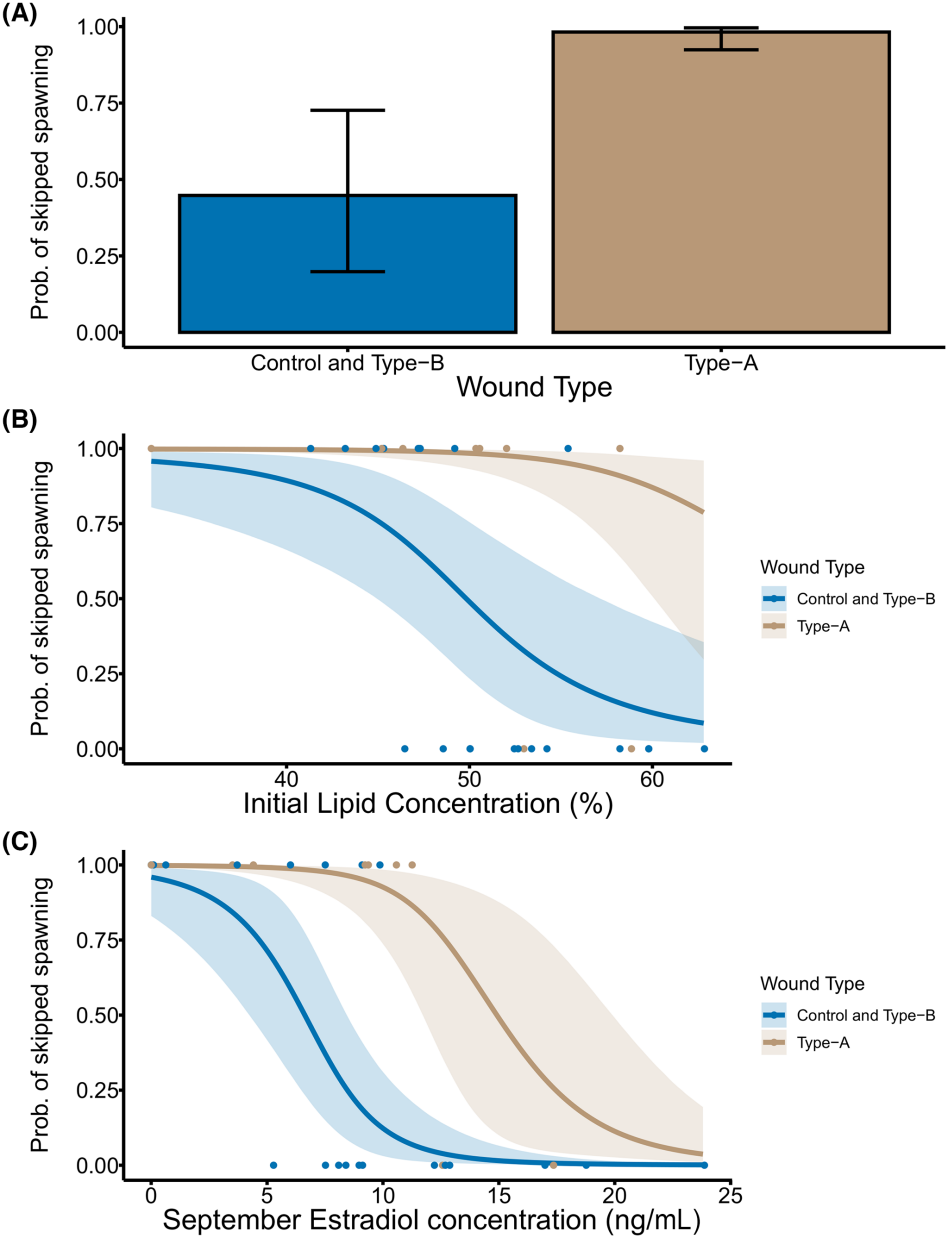
The influence of parasitism (**A**), initial lipid concentration (**B**), and September estradiol concentration (**C**) on the probability of skipping spawning for siscowet lake charr. Each panel shows the relationship between the probability of skipping spawning and the indicated variable calculated from the posterior chains of the most parsimonious model with all other variables held at their average values. For the influence of parasitism (**A**), the height of the bar indicates the posterior mean, and error bars represent 90% credible intervals. For the influence of initial lipid concentration (**B**) and September estradiol concentration (**C**), lines indicate the calculated probability of skipping spawning over a range of values for the indicated variable for type-A wounded and type-B wounded/control treatments, shaded areas indicate 90% credible intervals, and dots are observations of spawning.

Siscowet lake charr had more variable egg production than leans as a large proportion of siscowets skipped spawning (54%). A total of 78% (seven of nine) of siscowets with type-A wounds, 30% (3 of 10) with type-B wounds, and 50% (5 of 10) control siscowets skipped spawning. Parasitism heavily influences the likelihood of skipping spawning, and muscle lipid concentration prior to parasitism and plasma E2 concentration in September also play a role. The most parsimonious model estimating the likelihood of skipped spawning for included the presence of a type-A wound, initial muscle lipid concentration, and plasma E2 concentration in September (Supplementary Table S1). The odds of skipping spawning for siscowets with type-A wounds are 292 times higher (90%CrI = 11.24–14144.26) than control and type-B wounded siscowets (Fig. 4A). Every 1% increase in initial muscle lipid concentration decreased the odds of skipping spawning by 24% (90%CrI = 0.61–0.92; Fig. 4B). For every 1 ng/mL increase in September plasma E2 concentration there was a 50% (90%CrI = 0.31–0.73) decrease in the odds of skipping spawning (Fig. 4C). For the average siscowet in our sample, the probability of skipping spawning is 98% following parasitism with a type-A wound compared to only 17% when the siscowet was unparasitized or parasitized with a type-B wound. For the effect of muscle lipid concentration, a 10% decrease in initial muscle lipid increases the probability of skipping spawning by 52%. Similarly, a reduction in E2 concentration by 2 ng/mL increases the probability of skipping spawning from 24 to 45% for the average siscowet in our sample. One model, adding the presence of a type-B wound as an additional parameter, had a DIC value within 2 of the best performing model (Supplementary Table S1). However, in this model the 90%CrI for the type-B parameter overlapped with zero.

### Milt concentration

For male lean lake charr, parasitism did not influence milt sperm cell concentration, but the change in muscle lipid in the year following parasitism did (Supplementary Table S1). Every 1% increase in lipid corresponded with a decrease of 0.28 (90%CrI = −0.44 to −0.12) billion sperm cells per mL of milt. A lean gaining 1.8% muscle lipid in the year following parasitism (average change in our sample) would have a milt concentration of 5.32 billion sperm cells per mL, while one with no change in muscle lipid would have 5.83 billion sperm cells per mL. A model including the presence of a type-A wound in addition to change in muscle lipid had a DIC value within 2 of the best performing model (Supplementary Table S1), however the 90%CrI for the type-A parameter included zero.

**Fig. 5:**
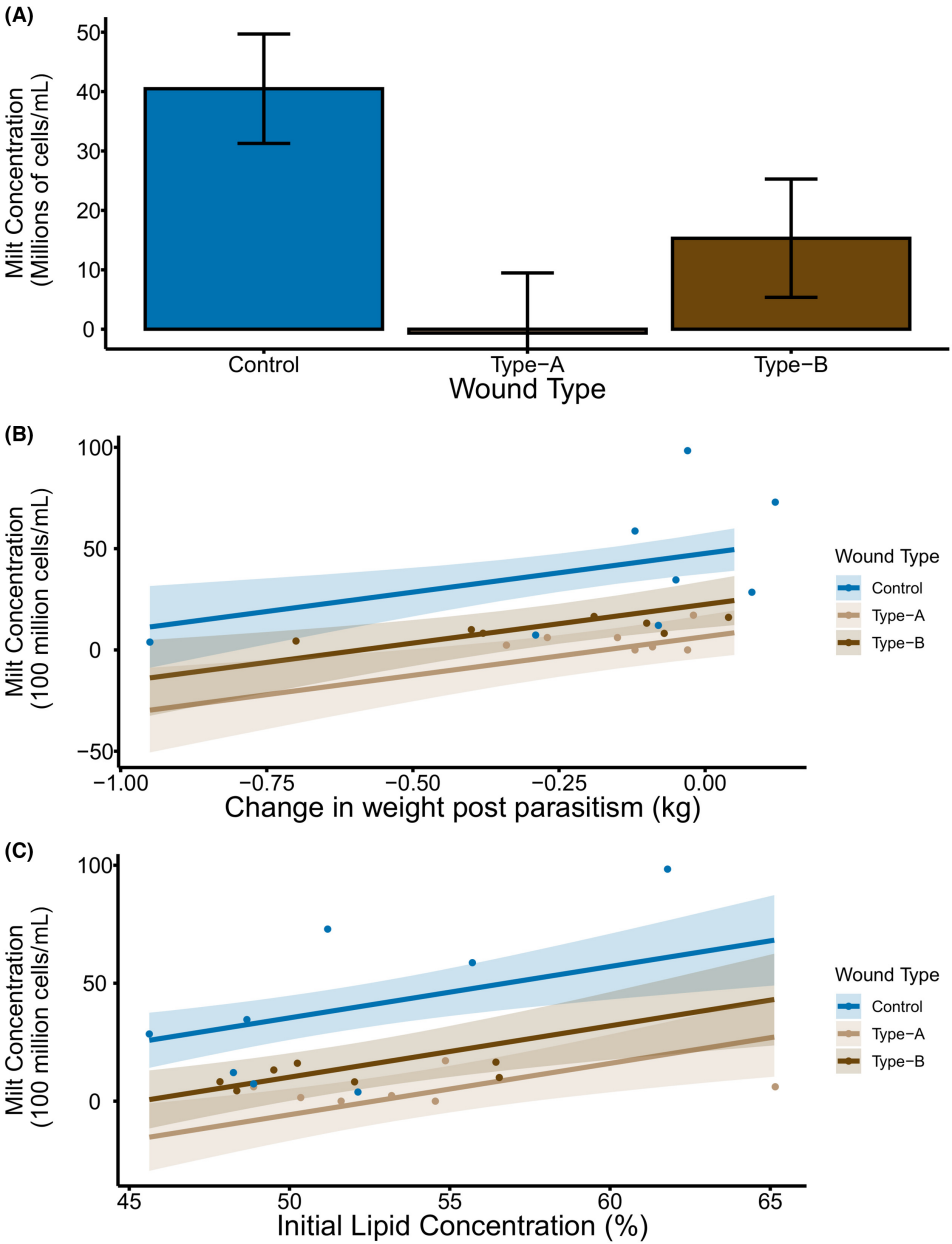
The influence of parasitism (**A**), change in weight following parasitism (**B**), and initial lipid concentration (**C**) on milt sperm cell concentration for siscowet lake charr. Each panel shows the relationship between milt sperm cell concentration and the indicated variable calculated from the posterior chains of the most parsimonious model with all other variables held at their average values. For the influence of parasitism (**A**), the height of the bar indicates the posterior mean, and error bars represent 90% credible intervals. For the influence of the change in weight following parasitism (**B**) and initial lipid concentration (**C**), lines indicate the calculated milt sperm cell concentration over a range of values for the indicated variable for type-A, type-B, and control treatments, shaded areas indicate 90% credible intervals, and dots are observed values. The location of observed values on the plot does not account for the influence of other variables.

For siscowet lake charr, milt concentration was influenced by parasitism, the change in weight during the year following parasitism, and muscle lipid concentration prior to parasitism (Supplementary Table S1). Parasitism leading to a type-A wound corresponded with a decrease of 4.11 (90%CrI = −5.64 to −2.58) billion sperm cells per mL of milt. Parasitism leading to a type-B wound corresponded with a decrease of 2.52 (90%CrI = −4.02 to −1.02) billion sperm cells per mL of milt. A 1 kg increase in weight in the year following parasitism was associated with an increase of 3.82 (90%CrI = 1.27–6.36) billion sperm cells per mL of milt, and a 1% increase in muscle lipid concentration prior to parasitism corresponded with an increase of 0.22 (90%CrI = 0.08–0.35) billion sperm cells per mL of milt (Fig. 5). With all other parameters held at their averages, an unwounded siscowet would have 4.04 billion sperm cells per mL, a type-A wounded siscowet would have −0.07 billion sperm cells per mL of milt, and a type-B wounded siscowet would have 1.53 billion sperm cells per mL of milt (Fig. 5A).

### Embryo survival

**Fig. 6:**
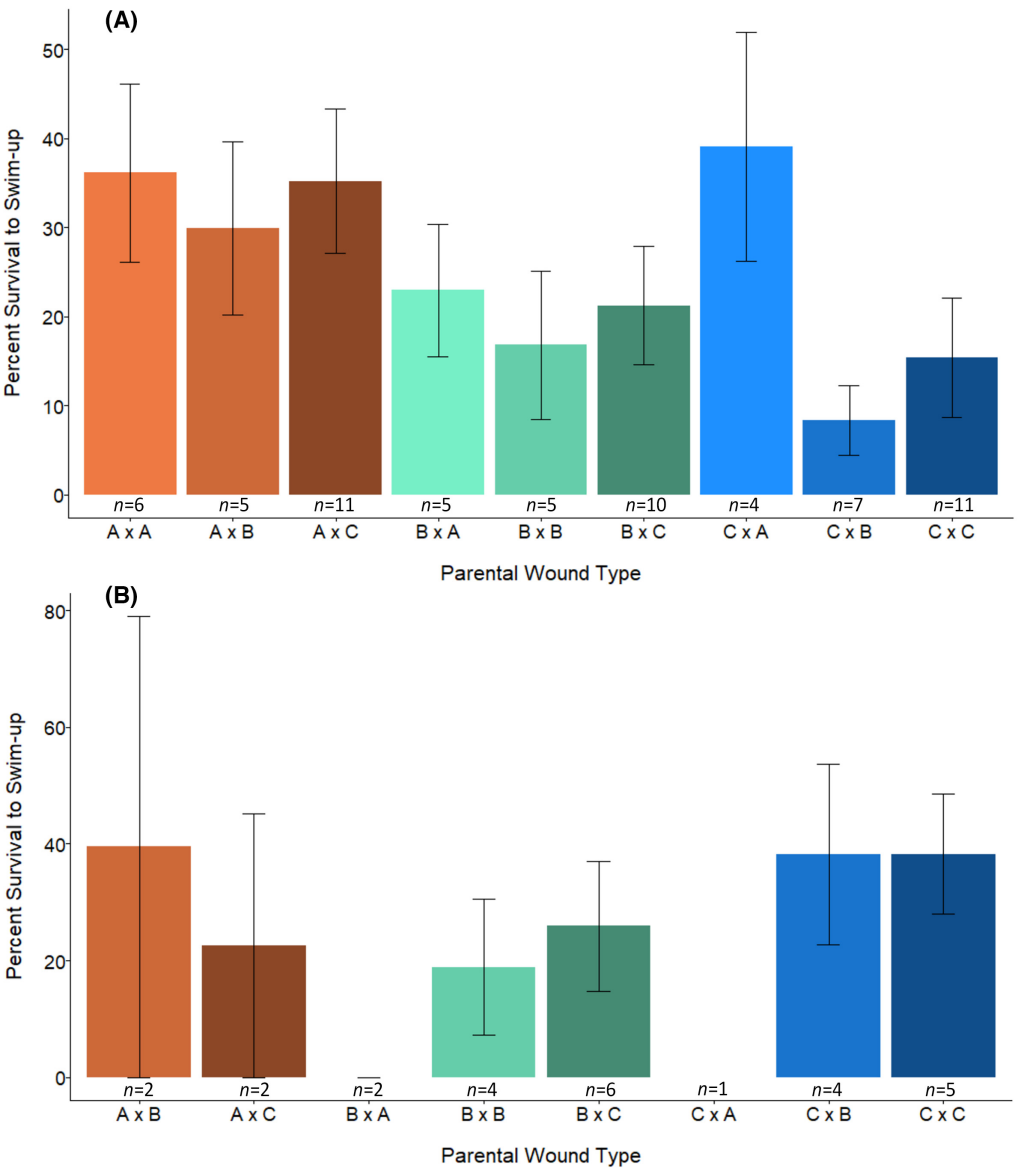
Embryo survival to swim-up for lean (**A**) and siscowet (**B**) lake charr by parental wound type. Bars indicate mean % survival for each group. Error bars indicate standard error of the mean, and sample sizes are included below each bar. Parental wound types are indicated as follows (female × male). A refers to type-A, B refers to type-B, and C refers to control. For example, “A x C” indicates eggs from a type-A wounded female and milt from a control male were used in the cross.

Because of the additional categories created during reproductive crosses and because a large proportion of siscowet lake charr skipped spawning, sample sizes for assessing embryo survival are too small for robust statistical analysis. Therefore, we only assessed these results qualitatively. Although there was considerable variation in embryo survival for siscowet lake charr, it was notable that no eggs fertilized by males with type-A wounds were viable (Fig. 6B). This trend was not present for leans (Fig. 6A).

## Discussion

We found that the response to sea lamprey parasitism differed between siscowet and lean lake charr and these differences, with few exceptions, matched the expectations laid out in our life history conceptual model (Table 1). Severely parasitized lean lake charr slightly increased their reproductive effort and maintained growth and energy storage, consistent with expectations based on life history given that leans are less likely to survive parasitism and have shorter lifespans than siscowet lake charr. Siscowets ceased reproduction almost completely following severe parasitism and showed evidence of altered energy storage, consistent with a strategy that favors maximizing long-term reproductive success at the expense of current reproduction. These findings suggest that life history can be used to generalize the response to sea lamprey parasitism. Our modeling approach also allowed us to identify other co-variates that were critical to explaining the changes we observed and allowed for these critical factors to be accounted for when examining the influence of parasitism. Without this approach, the influence of parasitism may have been obscured. We found that sex differences were important for understanding changes in HSI, and muscle lipid concentration prior to parasitism was critical for understanding reproduction and the relative influence of parasitism for siscowet lake charr.

### Growth

We expected that growth would be influenced by parasitism for both ecomorphs and that leans would divert energy from growth towards maintaining reproduction and siscowets would reduce growth to maintain energy storage. In many parasite–host interactions involving fish hosts, energy limitation from parasitism results in reductions of host growth (Britton et al. 2011; Godwin et al. 2017; Fjelldal et al. 2019). We did not find evidence that lean or siscowets were significantly altering growth in response to parasitism. One important consideration is that the age 12 lake charr used in this laboratory study were approaching their growth asymptotes and had small annual growth rates. As a result, changes in length or weight over time would likely be subtle, and thus difficult to detect. Additionally, evaluations of wild lake charr have found evidence of faster growth rates following severe parasitism (Smith et al. 2016), which could make identifying short-term reductions in growth difficult. Preliminary studies with younger fish suggest that there may be an immediate short-term reduction in growth following parasitism, but growth resumes in the long-term (Supplementary Fig. S1).

### Energy storage

We predicted that following parasitism, leans would reduce energy allocation towards storage while siscowets would maintain storage energy allocation. Because leans have fewer opportunities to reproduce over their lives than siscowets, we would expect them to prioritize reproduction over energy storage. However, we did not observe any alteration to muscle lipid concentration or HSI for lean lake charr resulting from parasitism. Similarly, siscowets did not significantly alter muscle lipid concentration following parasitism. Although siscowets have high muscle lipid content that could be available to mobilize following parasitism, lipid storage likely plays an important role in reducing the costs of maintaining neutral buoyancy at the depths they inhabit (Henderson and Anderson 2002; Goetz et al. 2014). Lipid storage is also hypothesized as an important siscowet life history strategy for building energy reserves sufficient for reproduction (Goetz et al. 2014), similar to Northeast Arctic cod (Skjæraasen et al. 2012). Given the functional role of muscle lipids for siscowet lake charr, parasitism effects on storage are more likely to be expressed in the HSI. For siscowets, HSI was influenced by an interaction between parasitism and sex. Sex differences in HSI have been well-documented in lake charr (Goetz et al. 2017) and are likely underpinning the differential response to parasitism. Male siscowet HSI is seasonally consistent, and therefore, the reductions in HSI we observed following severe parasitism (Fig. 2) is likely an indication of energy limitation. Female siscowet HSIs vary seasonally as liver weight is influenced by vitellogenin production during gamete development with higher HSI in the summer than in fall when reproduction is occurring (Goetz et al. 2017). We sampled HSI in late summer when female lake charr are beginning to ramp up gamete development for reproduction. The higher HSI values observed in type-A wounded female siscowets could be an indication that they are not mobilizing energy for reproduction. Alternatively, wild lake charr that skip spawning have been found to have lower HSI values than spawning lake charr (Sitar et al. 2014). Because a considerable number of control and type-B wounded siscowets also skipped spawning (see below) any effect of parasitism on HSI might be obscured. Also possible is that siscowets that skip spawning following type-A parasitism may be allocating energy towards reproduction differently than siscowets that skip spawning without the major stressor. Lake charr that skip spawning have been observed to undergo normal gonadal development until August where the maturation process stops and oocyte degeneration and resorption begins (Goetz et al. 2011; Sitar et al. 2014). Further work is necessary to identify the specific mechanisms at play.

### Reproduction

We expected female leans would maintain their reproductive effort while siscowets would divert energy away from reproduction to maximize future reproductive success. Female leans parasitized with type-A wounds produced more eggs than control and type-B wounded fish. An increased investment in egg production following severe parasitism makes sense because leans have fewer opportunities to reproduce over their lifespan (Chu and Koops 2007) and are more likely to die following parasitism than siscowets (Horns et al. 2003; Sitar et al. 2008). Therefore, reproductive success is maximized by investing in reproduction in the short-term. The less severe type-B parasitism was not sufficient to elicit an increase in reproductive investment. This is not surprising as mortality from type-B wounds are less frequent (Eshenroder and Koonce 1984) and the optimal life history strategy would not favor short-term reproduction. Surprisingly, the increased investment in reproduction following type-A parasitism did not result in any observed adverse trade-offs for growth and storage in lean lake charr. Despite the lack of observed trade-offs, it is possible that physiological processes we did not measure (such as lifespan) were influenced.

For female siscowet lake charr, the most striking influence on reproduction was the increased incidence of skipped spawning following severe parasitism. The increased odds of skipping spawning (292 times greater) for type-A wounded females was particularly large considering spawning was assessed approximately 1 year following a relatively brief parasitism event. Similar observations of skipped spawning occurring months after an acute stressor has been observed in polar cod (*Boreogadus saida*) exposed to burned oil residues (Bender et al. 2018). Skipping spawning is common in many long-lived fish species that rely on energy reserves to support gamete development or experience energy limitation (Rideout and Tomkiewicz 2011), and has been well-documented in siscowet lake charr (Goetz et al. 2011; Sitar et al. 2014). Our results largely align with observations of wild lake charr in Lake Superior. In southern Lake Superior, 58% of siscowet lake charr were observed to skip spawning (Sitar et al. 2014). The control and type-B wounded siscowets in our laboratory study skipped spawning at rates of 30% and 50% respectively, while 78% of type-A wounded siscowets skipped spawning. The likelihood of skipping spawning also depended on lipid concentration prior to parasitism and plasma E2 concentrations in September (when plasma E2 peaks for lake charr; Foster et al. 1993). Siscowets maintain high muscle lipid reserves and differ considerably in energy processing and storage dynamics compared to leans (Goetz et al. 2014; Sitar et al. 2020). These differences are heritable and are likely an adaptation for accumulating sufficient energy until a threshold is reached and reproduction proceeds. Our results make sense in this context as the energy limitations presented by severe sea lamprey parasitism would compete with the ability to accumulate energy for reproduction. Muscle lipid concentration prior to parasitism influenced skipped spawning, and further suggests that there is some baseline rate of skipped spawning that depends on a lipid storage threshold—parasitism demands a greater stored lipid requirement for successful reproduction. The presence of September E2 concentrations in the best performing model is consistent with observations of reduced plasma E2 concentrations in other skip spawning fish (Skjæraasen et al. 2009; Pierce et al. 2017). Estradiol modulates hepatic production and gonadal uptake of the egg-yolk protein vitellogenin (Tyler and Sumpter 1996), and is therefore, critical for gonadal development and may be useful as an early biomarker of skipped spawning.

We evaluated effects on male reproduction by assessing influences on milt concentration. For lean males, parasitism status did not have an effect. The change in muscle lipid concentration in the year following parasitism best predicted milt concentration and indicates that the more energy a lean invests in storage (regardless of parasitism status), the less energy is available to invest in milt production. The lack of parasitism effects matches our expectations that leans will maintain reproductive output following parasitism so that short-term reproductive success is maximized. While our results do not indicate evidence of a parasitism-driven change in reproductive output, they may highlight a life history trade-off between energy storage and reproduction for male leans.

**Fig. 7:**
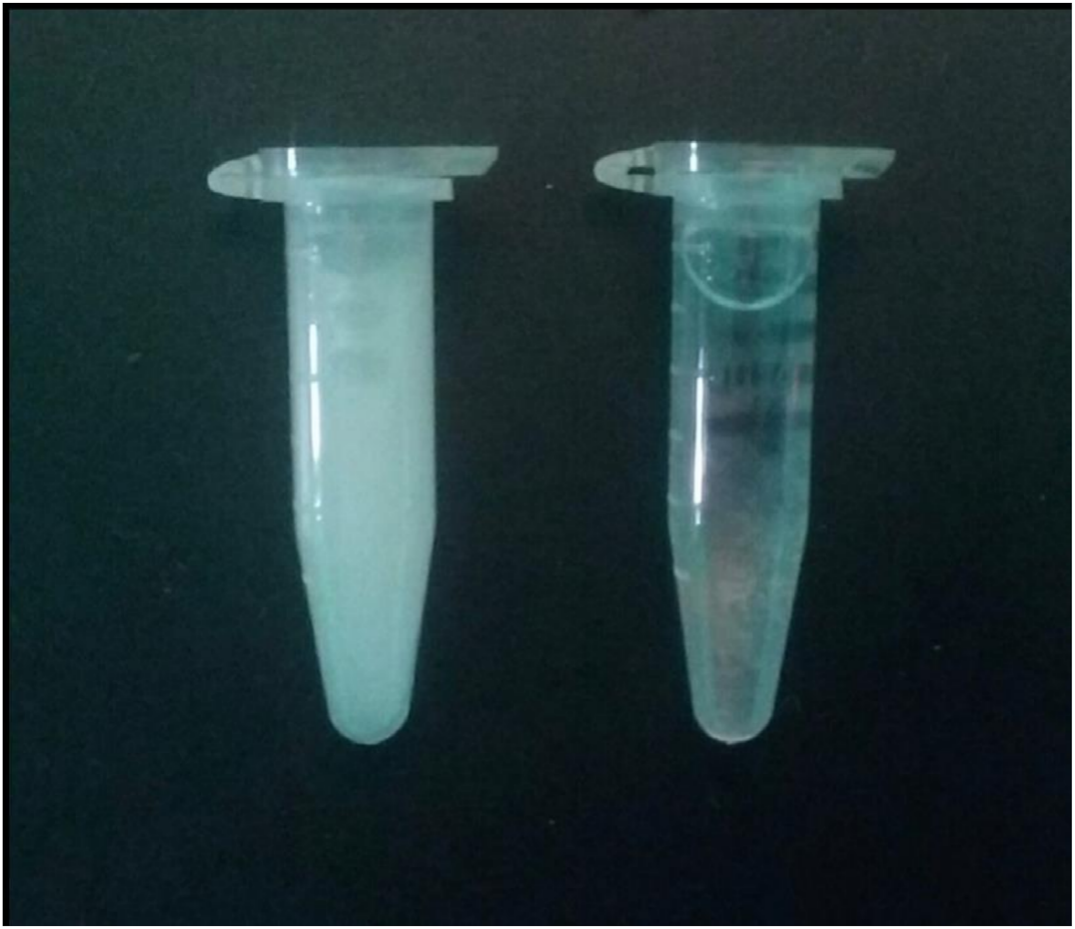
Examples of milt sampled from a siscowet control male (left) and a type-A wounded male (right).

Male siscowet milt concentration was influenced by parasitism status change in weight in the year following parasitism, and initial muscle lipid concentration. Interestingly, siscowet milt concentration was the only endpoint where type-B parasitism was distinguished from control fish. Effects on milt concentration matched our expectations based on the severity of parasitism with type-B wounds being associated with a smaller reduction in milt concentration than type-A wounds. Similar to our observations with female egg production and skipped spawning, we expected male siscowets to also reduce reproductive effort following parasitism to maximize future reproductive success. There were striking visual differences in milt with most of the parasitized males with type-A wounds having nearly transparent milt compared to the milky white color of control male milt (Fig. 7). The milt produced by males with type-A wounds was nonviable, and no eggs fertilized with this milt survived (Fig. 6B). This trend was not present for leans (Fig. 6A). These dramatic changes in milt quality were surprising given these effects were observed approximately 1 year after a brief (4 day) sea lamprey attack. In lake superior, siscowets have been observed with very low GSI and no signs of spermatogenesis during normal spawning (Goetz et al. 2011), suggesting that some male siscowets skip spawning in a similar manner to females. Although we did not observe males with no sperm cells in their milt, sperm counts were very low and the collected milt failed to successfully fertilize eggs, effectively skipping spawning.

Gamete production is generally more energetically demanding for females than males, so we expected to see more subtle effects on male reproduction. However, similar severe effects on male reproduction have been observed in closely related Arctic charr with high macroparasite loads (*Salvelinus alpinus*; Skarstein et al. 2001). Sperm cells are antigenic, and part of the role of testosterone in male fish is to suppress the autoimmune response that would otherwise attack sperm cells (Hillgarth et al. 1996). Given the considerable cross-talk between the immune system and the hypothalamus–pituitary–gonadal axis (Segner et al. 2017), it is possible that testosterone and other androgens produced during sperm development would suppress immune function and inhibit the fish's ability to sufficiently cope with parasitism. If so, this process may provide a further incentive for male siscowets to forgo reproduction. Similar to female siscowets, muscle lipid concentration prior to parasitism was predictive of milt. We also hypothesize that this is due to the life history of siscowets, whereby stored lipid reserves are required for reproduction independent of parasitism. This is supported by findings that wild siscowets with higher energy storage (as measured by HSI) were more likely to spawn (Sitar et al. 2014). The change in weight in the year following parasitism was also positively associated with milt concentration in our model. We found that change in weight was not significantly associated with parasitism status, but instead we suggest this effect is simply due to the positive relationship between size and reproductive output.

The finding that parasitism increases the incidence of skipped spawning for male and female siscowets has important implications for life history theory. Skipped spawning is thought to be an adaptation by some fish species to energy resource limitation, density-dependence, or suboptimal environmental conditions (Rideout et al. 2005). Under these conditions, skipping spawning allows for energy reserves to be built and future reproductive success to be maximized when energetic and environmental conditions are likely more favorable (Rideout et al. 2005; Rideout and Tomkiewicz 2011). Our findings that muscle lipid reserves measured 1 year before spawning were an important factor for estimating the likelihood of skipping spawning independent of parasitism support this idea. The relatively high rate of skipped spawning in control fish (50%) was still surprising, given the fact that food was abundantly available and energy resource limitation due to a lack of food would be unlikely. One explanation is that skipped spawning is not solely a response to environmental conditions, but also a programed response to adaptations siscowets have for living in consistently low water temperatures that leave less energy available to allocate towards reproduction (Goetz et al. 2014). Thus, skipped spawning naturally occurs at some baseline rate for siscowet lake trout, and environmental factors that limit energy storage increase the likelihood of skipping (Goetz et al. 2021). Another possibility is that under high food availability, skipped spawning may be more common as individuals can opportunistically increase growth and take advantage of the future benefits of increased body size for reproductive output when food availability may be lower (Jørgensen et al. 2006; Rideout and Tomkiewicz 2011). It is likely that all of these factors play a role in skipped spawning, and sea lamprey parasitism simply increases the likelihood that a fish will skip due to energy limitation.

### Study limitations

There are several important limitations with our study that should be considered when comparing the physiological responses we observed under laboratory conditions to lake charr in the field. One limitation is that lake charr in our laboratory conditions were provided with ample easy-to-access food. If parasitism had an influence on feeding behavior or ability to capture prey fish, we likely would not have observed these effects under laboratory conditions. The presence of ample food under laboratory conditions could obscure effects on growth, energy storage, and reproduction that would result from an inability to capture sufficient prey in the wild. An additional limitation is that we removed sea lamprey from hosts after 4 days of feeding to prevent lethal parasitism. In the wild, sea lamprey feeding duration is much more variable, and appears to depend on water temperature and sea lamprey body size (Swink 1993, 2003).

The environmental conditions provided in our study also do not necessarily match conditions experienced by wild lake charr. Siscowet lake charr in particular are adapted to live in deep water and experience high pressure (up to 41 atmospheres), low light, and relatively constant water temperatures (4°C; Sitar et al. 2008). At times, however, the siscowet ecomorph will reside near the surface for long periods (up to a month) and display periodic extreme vertical movements from the bottom to the surface (Jasonowicz et al., submitted for publication) exposing them to lower pressures, higher light intensities, and warmer temperatures. The conditions provided by the raceway environment were generally warmer (6.8–8.3°C), lower pressure, and brighter than the siscowet ecomorph would typically experience while residing in deep water habitats. Consequently, there could be an influence on the response to parasitism or environmental cues important for the physiological functions we observed. For example, the lower water temperatures experienced by siscowet lake charr in the wild is often attributed to their higher survival following sea lamprey parasitism (Bence et al. 2003; Sitar et al. 2008), and therefore, the warmer water temperatures in our study may result in more severe consequences for siscowet lake charr than would be expected in the wild.

Despite these limitations, our observations are largely mirrored in observations of field caught lake charr. For example, the rates of skipped spawning we observed for siscowet lake charr are in-line with observations of wild lake charr in southern Lake Superior (Sitar et al. 2014). Wild siscowet lake charr that had experienced parasitism did not display altered growth trajectories, similar to our observations (Smith et al. 2016). Nevertheless, the differences in laboratory versus natural conditions should be considered when extrapolating these results to inform the management of wild lake charr.

## Conclusion

Our results indicate that siscowet and lean lake charr differ in their response to parasitism, and these different responses largely match our expectations given the different life histories of these two ecomorphs. Because lean lake charr are relatively short lived (Chu and Koops 2007), reach reproductive maturity faster (Sitar et al. 2014), and are less likely to survive parasitism (Horns et al. 2003; Sitar et al. 2008), maximizing short-term reproduction makes sense. For siscowet lake charr, forgoing reproduction so that energy can be stored for future reproduction is more advantageous as it maximizes lifetime reproduction in the long run. This study is the first time we are aware of that these life history trade-offs have been empirically examined in the context of sea lamprey parasitism.

Currently, the sub-lethal effects of sea lamprey parasitism are not considered in lake charr management plans or population models, and wounded fish are assumed to reproduce and function like unparasitized lake charr. This means that many of the potential consequences of sea lamprey parasitism are not accounted for when informing the management of the fishery. For example, records of sea lamprey wounds observed on lake charr captured during biological monitoring surveys are used as a standardized metric of sea lamprey damage in the Great Lakes (Treska et al. 2021). These marking rates inform sea lamprey control efforts and estimated rates of lake charr mortality. A target of fewer than 5 A-I through A-III marks per 100 lake charr over 533 mm in length (2 A-I marks per 100 lake charr over 432 mm for Lake Ontario) was developed based on the maximum level of sea lamprey-induced mortality that fisheries managers were willing to accept (Treska et al. 2021). If the sublethal effects of sea lamprey parasitism were also considered when setting this target, the target marking rate may need to be lowered. Our research suggests the inclusion of the sublethal effects of parasitism would help refine and improve these targets.

## Authors’ contributions

Conceptualization: C.A.M, T.J.F., and F.W.G; methodology: G.F., T.J.F., F.W.G., and C.A.M; formal analysis: T.J.F; investigation: T.J.F., G.F., and C.A.M.; resources: C.A.M., F.W.G., and G.F.; writing-original draft preparation: T.J.F.; writing-review and editing: C.A.M., F.W.G., G.F., and T.J.F.; visualization: T.J.F.; supervision: C.A.M., T.J.F., and G.F.; project administration: C.A.M., T.J.F., and G.F.; and funding acquisition: C.A.M.

## Supplementary Material

icac001_Supplemental_File

## Data Availability

Data will be uploaded to a Dryad repository upon acceptance.
