# Supplementary material for: The Influence of Life History on the Response to Parasitism: Differential Response to Non-Lethal Sea Lamprey Parasitism by Two Lake Charr Ecomorphs
Source: Integr Comp Biol. 2022 Jan 13;62(1):104–20. doi: 10.1093/icb/icac001 (PMC9375137; doi:10.1093/icb/icac001)
Supplement: icac001_Supplemental_File [file icac001_supplemental_file.docx]

**Supplementary Materials:**

Table S1. Model performance of the top 5 candidate models for each of our endpoints of interest as ranked by the deviance information criterion (DIC).

| Model Type | Ecomorph | Parameters | DIC | Δ DIC |
| --- | --- | --- | --- | --- |
| Change in length | Siscowet | LipInit | 397.6 | 0.0 |
|  |  | sex | 397.7 | 0.1 |
|  |  | woundA | 398.5 | 0.9 |
|  |  | woundA + LipInit | 399.0 | 1.4 |
|  |  | woundA + woundB | 399.6 | 2.0 |
|  |  |  |  |  |
|  | Lean | woundA | 362.6 | 0.0 |
|  |  | LipInit | 363.5 | 0.9 |
|  |  | woundA + sex | 364.2 | 1.6 |
|  |  | woundA + LipInit | 364.2 | 1.6 |
|  |  | woundA + woundB | 364.7 | 2.1 |
|  |  |  |  |  |
| Change in weight | Siscowet | woundA + woundB | 30.3 | 0.0 |
|  |  | woundA | 31.6 | 1.3 |
|  |  | woundA + woundB + sex | 32.6 | 2.3 |
|  |  | woundA + woundB + LipInit | 32.6 | 2.3 |
|  |  | LipInit | 33.3 | 3.0 |
|  |  |  |  |  |
|  | Lean | LipInit | -9.3 | 0.0 |
|  |  | woundA | -9.1 | 0.2 |
|  |  | woundA + woundB | -7.5 | 1.8 |
|  |  | woundA + LipInit | -7.0 | 2.3 |
|  |  | woundA + sex | -6.8 | 2.5 |
|  |  |  |  |  |
| Egg production | Siscowet | woundA + LipInit | 156.2 | 0.0 |
|  |  | LipInit | 156.5 | 0.3 |
|  |  | woundA + Esep + LipInit | 156.5 | 0.4 |
|  |  | LipInit + Esep | 156.9 | 0.8 |
|  |  | woundA + woundB + LipInit | 157.9 | 1.8 |
|  |  |  |  |  |
|  | Lean | woundA + deltL | 131.4 | 0.0 |
|  |  | deltL | 134.7 | 3.3 |
|  |  | deltWt | 137.3 | 6.0 |
|  |  | deltLip | 138.1 | 6.7 |
|  |  | woundA + deltWt | 138.4 | 7.0 |
|  |  |  |  |  |
| Skipped spawning | Siscowet | woundA + LipInit + Esep | 20.1 | 0.0 |
|  |  | woundA + woundB + LipInit + Esep | 21.4 | 1.2 |
|  |  | woundA + woundB + Esep | 26.6 | 6.5 |
|  |  | LipInit + Esep | 28.2 | 8.0 |
|  |  | deltWt + Esep + LipInit | 28.3 | 8.1 |
|  |  |  |  |  |
| Change in muscle lipid | Siscowet | sex | 319.7 | 0.0 |
|  |  | woundA + sex | 321.9 | 2.2 |
|  |  | woundA + woundB + sex | 322.6 | 2.9 |
|  |  | woundA | 330.8 | 11.1 |
|  |  | woundA + woundB | 331.5 | 11.8 |
|  |  |  |  |  |
|  | Lean | LipInit | 392.2 | 0.0 |
|  |  | woundA | 393.1 | 0.9 |
|  |  | woundA + woundB | 394.7 | 2.5 |
|  |  | woundA + LipInit | 394.5 | 2.3 |
|  |  | woundA + sex | 395.2 | 3.0 |
|  |  |  |  |  |
| Hepatosomatic index | Siscowet | woundA + female + woundA*female | 10.5 | 0.0 |
|  |  | female | 11.3 | 0.8 |
|  |  | woundA + woundB + female + woundA*female | 11.9 | 1.4 |
|  |  | woundA + LipInit + female + woundA*female | 13.0 | 2.5 |
|  |  | woundA + female | 13.5 | 3.0 |
|  |  |  |  |  |
|  | Lean | female | -8.8 | 0.0 |
|  |  | woundA + female | -6.4 | 2.4 |
|  |  | woundA + female + woundA*female | -4.3 | 4.5 |
|  |  | woundA + woundB + female | -3.8 | 5.0 |


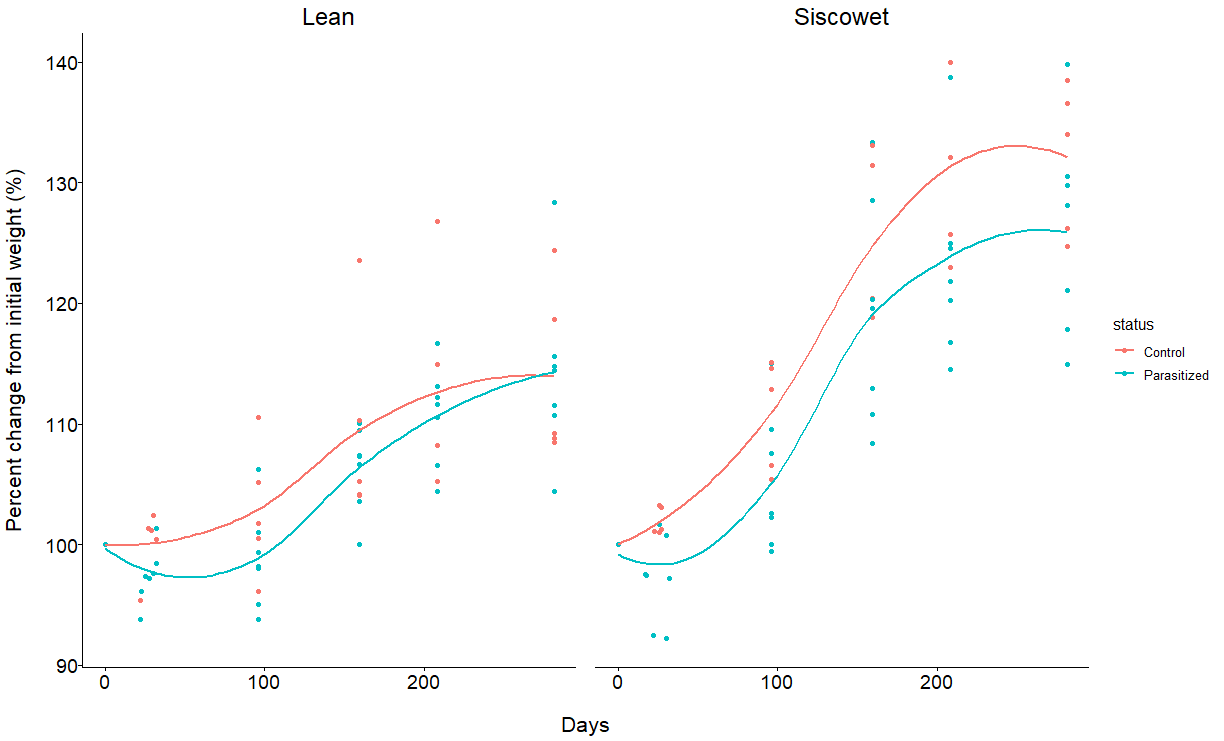


Figure S1. Percent change in weight for 5-year-old lean and siscowet lake charr from a companion study. A group of younger siscowet and lean lake charr were parasitized by sea lamprey following the same procedures outlined in the methods of this study. Wet weight was monitored monthly. Change in weight is presented relative to weights just prior to parasitism. Boxes on the X-axis indicate the time that parasitism trials took place. Trend lines indicate loess smoothed regressions for parasitized and control categories. Sample sizes for leans were: parasitized n=5, control n=7. Sample sizes for siscowets were: parasitized n=7, control n=5.


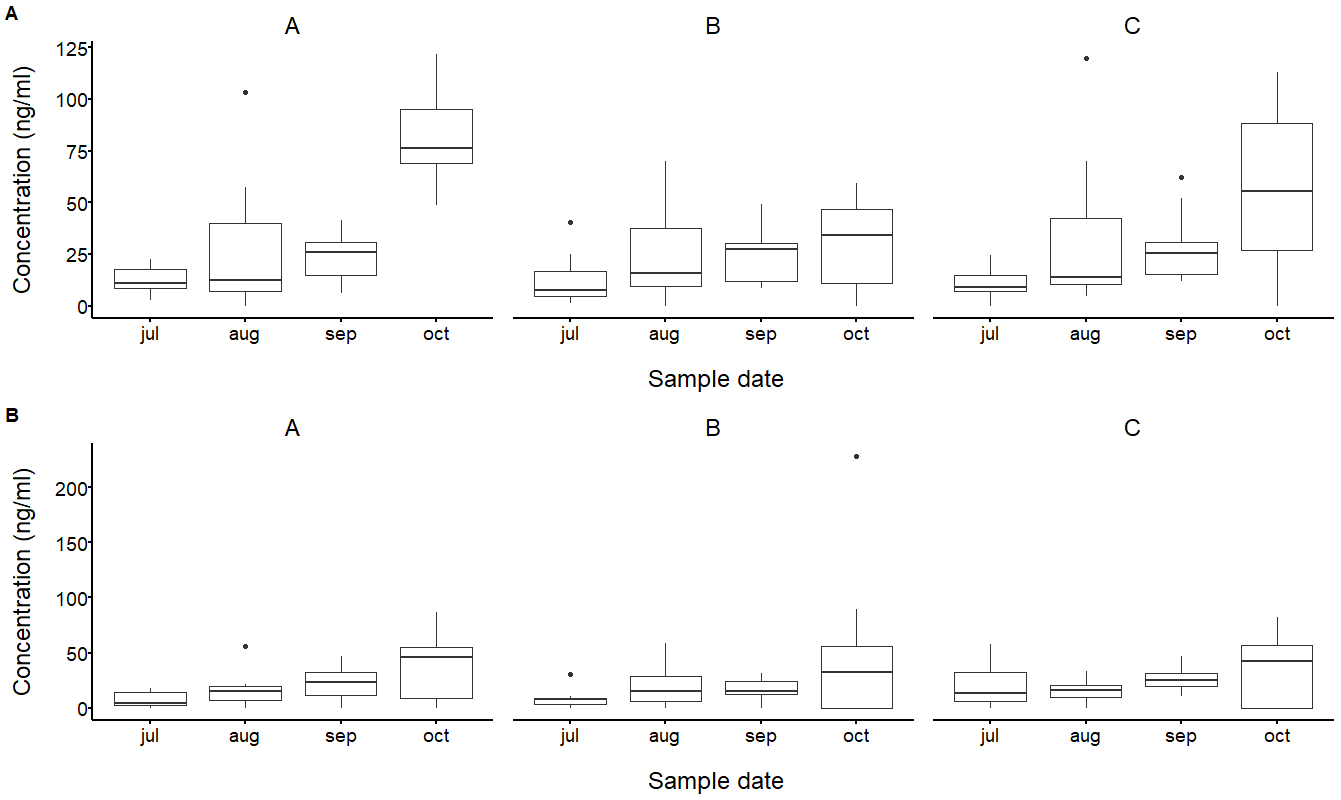


Figure S2. Testosterone (T) profiles for female lean (A) and siscowet (B) lake charr by parasitism status. Boxes indicate interquartile range, thick horizontal lines indicate medians, vertical lines indicate highest and lowest values, and dots indicate outliers. Type-A wounded, type-B wounded, and control fish profiles are indicated by A, B, and C respectively.


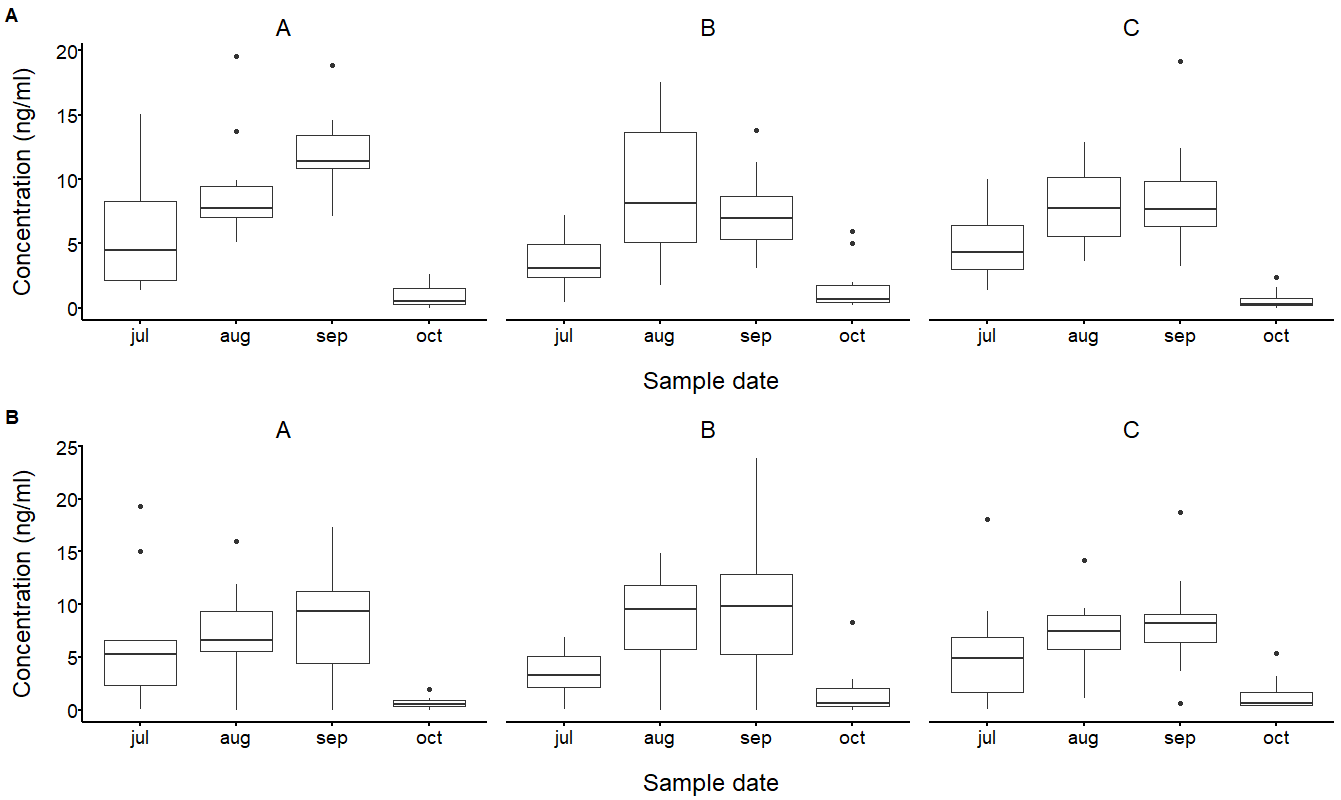


Figure S3. Estradiol (E2) profiles for female lean (A) and siscowet (B) lake charr by parasitism status. Boxes indicate interquartile range, thick horizontal lines indicate medians, vertical lines indicate highest and lowest values, and dots indicate outliers. Type-A wounded, type-B wounded, and control fish profiles are indicated by A, B, and C respectively.
